# Voltammetric Electrochemical Sensor for Phylogenetic Study in *Acer* Linn.

**DOI:** 10.3390/bios11090323

**Published:** 2021-09-08

**Authors:** Qingwei Zhou, Kewei Liu, Xiaolong Li, Yonghua Gu, Yuhong Zheng, Boyuan Fan, Weihong Wu

**Affiliations:** 1 College of Materials and Environmental Engineering, Hangzhou Dianzi University, Hangzhou 310018, China; zhouqw@hdu.edu.cn (Q.Z.); lxlr@hdu.edu.cn (X.L.); 191200023@hdu.edu.cn (B.F.); 2 School of Environment Science and Spatial Informatics, Xuzhou Campus, China University of Mining and Technology, Xuzhou 221116, China; 3 Zhejiang Huachuan Industrial Group Co., Ltd., Yiwu 322003, China; 4 Institute of Botany, Jiangsu Province & Chinese Academy of Sciences (Nanjing Botanical Garden, Mem. Sun Yat-Sen), Nanjing 210014, China; guyhua@cnbg.net (Y.G.); zhengyuhong@cnbg.net (Y.Z.)

**Keywords:** voltammetric sensor, electrochemical fingerprint, electroactive compounds, *Acer* Linn., pattern recognition

## Abstract

*Acer* Linn. is a highly divergent species morphology in the maple family (Aceraceae). It is one of the genera facing a very difficult taxonomic situation. The phylogeny of the genus and the taxonomic system under the genus remain unclear. The use of electrochemical fingerprints for plant phylogenetic study is an emerging application in biosensors. In this work, leaves of 18 species of *Acer* Linn. with an exo-taxa were selected for electrochemical fingerprint recording. Two different conditions were used for improving the data abundance. The fingerprint of all species showed a series of oxidation peaks. These peaks can be ascribed to the oxidation of flavonols, phenolic acids, procyanidins, alkaloids, and pigments in plant tissue. These electrochemical fingerprints can be used for the identification of plant species. We also performed a phylogenetic study with data from electrochemical fingerprinting. The phylogenetic tree of *Acer* is divided into three main clades. The result is in full agreement with *A. shangszeense* var. *anfuense*, *A. pictum* subsp. *mono*, *A. amplum*, *A. truncatum*, and *A. miaotaiense*, belonging to the subsection Platanoidea. *A. nikoense* and *A. griseum* were clustered together in the dendrogram. Another group that fits the traditional classification results is in the subsection Integrifolia.

## 1. Introduction

*Acer* Linn. belongs to the maple family (Aceraceae) and is a small deciduous or evergreen tree with more than 200 species. Fossil studies prove that the genus Maple originated in the Jurassic era and formed a truly modern species by the Pliocene epoch. During the long period of dispersal and natural evolution, *Acer* have retained their unique winged fruit morphology. However, many other morphological characteristics, such as bud scales, inflorescences, leaf, and fruit shapes, have undergone a high degree of variation, which makes systematic taxonomic studies of *Acer* very difficult. Pax divided the genus *Acer* into 4 subgenera and 14 groups after studying the morphological characteristics, geographic distribution, and fossil material information of the genus, which was later adjusted to 13 groups [1,2]. The classification system of Pax was revised by Rehder [3] and Koidzumi [4], successively. Phylogenetic investigation is a difficult task in the study of *Acer*. Traditional phylogenetic studies of the *Acer* mainly rely on morphological taxonomy, which affects the accuracy of the results because it is difficult to avoid the interference of environmental factors. Since the establishment of branching systematics, the method has been widely used for phylogenetic studies of *Acer*. Different studies were conducted to construct a branching phylogenetic tree of the *Acer* by studying the morphological characteristics of scale buds, inflorescences, leaves, seeds, pollen, etc. In recent years, with the rapid development of molecular biotechnology, DNA barcodes, such as internal transcribed spacer (ITS), matK, atpF-atpH, rbcL, ycf1, trnH-psbA, and trnL-trnF, have been widely used in the phylogenetic study of plants. For example, Ackerly and Donoghue [5] explored the evolutionary relationships of three morphological characters of *Acer* plants in relation to ITS sequence information. There is also some work that analyzed the phylogenetic relationships among some groups of the *Acer* using ITS sequences [6,7,8,9,10]. Due to the high homology of maple ITS sequences and the potential for homologous evolutionary incompleteness caused by long generations and hybridization, it is difficult to conduct taxonomic and phylogenetic studies of maples with a single ITS sequence.

Electrochemical fingerprinting is a technique for collecting electrochemically active substances in plant tissues. Since the electrochemical signal is proportional to the type and content of the substance, it can reflect the difference of electrochemically active substance profile in plants [11,12,13,14,15,16,17,18,19,20,21,22,23]. For example, Doménech-Carbó et al. [14] demonstrated the voltammetric fingerprints of *Asparagus* seeds can reflect the profiles of polyphenolic compounds (daidzein, ellagic acid, gallic acid, genistein, morin, quercetin, and rutin). Ren et al. [20] reported the electrochemical signal changes can be used for profiling hydrogen peroxide, nitric oxide, and pH in plants. This technique has been applied in recent years to the study of plant phylogeny and has become an alternative technique to provide evidence [24,25,26,27,28,29,30,31,32,33,34,35]. However, this electrochemical-based phylogeny has not been used for investigations of complex genus, such as those of the *Acer*. In this work, we tried to apply this technique to the study of the *Acer*. Eighteen species of *Acer* with an exo-taxa were selected for electrochemical fingerprint recording. The results of the obtained cluster analysis and the proposed claims were studied in comparison.

## 2. Materials and Methods

Leaves of *Acer shangszeense* var. *anfuense*, *A. pictum* subsp. *mono*, *A. amplum*, *A. truncatum*, *A. miaotaiense*, *A. nikoense*, *A. griseum*, *A. fabri* Hance var. *rubrocarpum*, *A. paxii*, *A. oblongum*, *A. laevigatum*, *A. buergerianum* var. *formosanum*, *A. cinnamomifolium*, *A. buergerianum*, *A. cordatum*, *A. fabri*, *A. sterculiaceum* subsp. *Franchetii*, *A. stachyophyllum* subsp. *betulifolium*, and *Dipteronia dyerana* were supplied by Nanjing Botanic Garden. All leaves were collected in June 2021. When collecting, only mature and healthy leaves were harvested. All samples were kept frozen before analysis. Ethanol, KH_2_PO_4_, K_2_HPO_4_, acetic acid, sodium acetate, and NaCl were analytical grade and used without further purification. The extraction process was conducted using ethanol and water as a solvent. A total of 0.1 M of PBS (pH 7.0) and ABS (pH 4.5) were used as a supporting electrolyte. All electrochemical fingerprint recordings were conducted using a CHI760 electrochemical workstation. A commercial glassy carbon electrode (Φ3 mm, GCE, Gaossunion Photoelectric Technology Co., Ltd.), an Ag/AgCl (3 M KCl) electrode, and a Pt wire electrode were used as the working electrode, reference electrode, and counter electrode, respectively. The GCE required in the experiment was polished on the wetted Al_2_O_3_ powder to the mirror surface, which was cleaned with secondary distilled water. Water and ethanol were directly used as solvents for extraction. Specifically, 10 mL of solvent was added into 2 g chopped plant tissue by 1 min grinding. A total of 0.1 M supporting electrolyte was then added for 3 min of sonication. Voltammetric fingerprint recording was conducted by a three-electrode system. Differential pulse voltammetry (DPV) was used for electrochemical recording. The scan range is 0–1.3 V (pulse amplitude: 50 mV; pulse width: 0.05 s; pulse period: 0.5 s).

A normalization process was conducted for all recorded electrochemical fingerprints, where the ratios between the current and the maximum peak current were obtained at different potentials. For the principal component analysis, we superimposed two sets of standardized electrochemical data and finally extracted three principal components. The taxonomic analysis was carried out using hierarchical clustering method based on fingerprint recorded in two conditions [36]. By using this algorithm, the data set is divided into different clusters by iteratively merging or splitting clusters based on a dendrogram. Clustering algorithms provide in a compact and graphical way information about the data to be classified. They can be agglomerative or divisive depending on the used procedure to create the dendrogram. Agglomerative clustering follows a bottom-up strategy in which, initially, each data point is assumed as a cluster, and then, iteratively, it merges the two most similar clusters in terms of an objective function until arriving at the final dendrogram. By contrast, divisive clustering follows an opposite approach. All data points are considered initially as one cluster, and then, iteratively, the selected cluster is partitioned into two new subclusters. In complete linkage clustering, the distance between two clusters is the distance between their closest or their two most distant data points.

## 3. Results and Discussion

**Figure 1:**
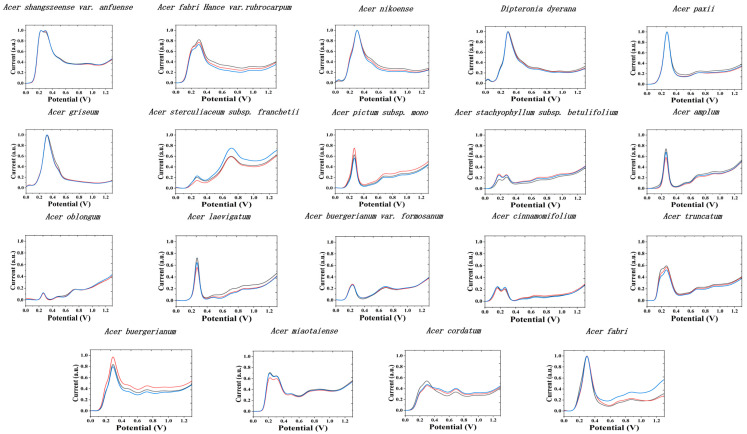
Electrochemical fingerprint of *A. shangszeense* var. *anfuense*, *A. pictum* subsp. *mono*, *A. amplum*, *A. truncatum*, *A. miaotaiense*, *A. nikoense*, *A. griseum*, *A. fabri Hance* var. *rubrocarpum*, *A. paxii*, *A. oblongum*, *A. laevigatum*, *A. buergerianum* var. *formosanum*, *A. cinnamomifolium*, *A. buergerianum*, *A. cordatum*, *A. fabri*, *A. sterculiaceum* subsp. *Franchetii*, *A. stachyophyllum* subsp. *betulifolium*, and *Dipteronia dyerana* after water extraction and recording in PBS (0.1 M, pH 7) condition.

**Figure 2:**
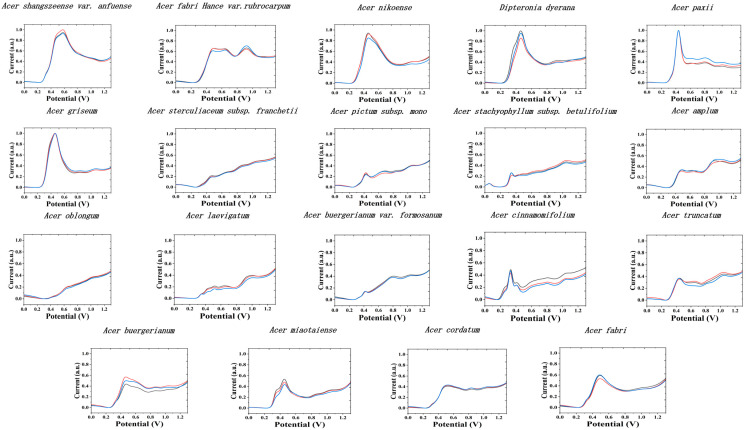
Electrochemical fingerprint of *A. shangszeense* var. *anfuense*, *A. pictum* subsp. *mono*, *A. amplum*, *A. truncatum*, *A. miaotaiense*, *A. nikoense*, *A. griseum*, *A. fabri Hance* var. *rubrocarpum*, *A. paxii*, *A. oblongum*, *A. laevigatum*, *A. buergerianum* var. *formosanum*, *A. cinnamomifolium*, *A. buergerianum*, *A. cordatum*, *A. fabri*, *A. sterculiaceum* subsp. *Franchetii*, *A. stachyophyllum* subsp. *betulifolium*, and *Dipteronia dyerana* after ethanol extraction and recording in ABS (0.1 M, pH 7) condition.

No surface modification of GCE was performed in our present work. This is because many commonly used nanomodified materials have electrocatalytic activity, which is very helpful in detecting a particular analyte. However, in the recording of electrochemical fingerprinting, this can lead to distortion of the signal. Therefore, nanomaterial modified electrodes are not the optimal choice in our work. Figure 1 shows the electrochemical fingerprints of all species recorded after water extraction and using PBS (0.1 M, pH 7) as the electrolyte. It can be seen that the differential pulse voltammetry (DPV) of all species showed a series of oxidation peaks. These peaks can be ascribed to the oxidation of flavonols, phenolic acids, procyanidins, alkaloids, and pigments in plant tissue [37,38,39,40]. The molecules oxidized at lower potentials are often small molecular-weight phenols [41]. It can be seen from the figure that each species exhibits a clear oxidation peak in the range of 0.0–0.4 V. Although it is not possible to give the specific molecules oxidized (many molecules with similar structures oxidize at similar potentials and overlapping each other), there is a positive correlation between the intensity of the fingerprint and the content of the molecules. We optimized the pH of the electrolyte. We chose electrolytes with different pH because different electrochemically active substances can participate in electrochemical reactions in different pH environments. We collected electrochemical fingerprints in order to fully reflect the profile of electrochemically active substances in plants. The results found that the fingerprint profile could only present limited information in alkaline environment. Acidic and neutral conditions fingerprint profiles can have richer information. Therefore, we selected acidic ABS and neutral PBS as electrolytes. The electrochemical fingerprints varied considerably among the different species. This is due to the fact that there are large differences at the genetic level between the different species and therefore the composition of the plant tissues [18]. However, some of these species have more similar fingerprint among themselves. For example, the DPV profiles of *A. stachyophyllum* subsp. *betulifolium* and *A. cinnamomifolium* are very similar. They also showed two characteristic peaks between 0.0–0.4 V. Although *A. stachyophyllum* subsp. *betulifolium* has a small peak over 1.0 V, this flat peak not obviously enough for identification. This represents that the substances involved in the electrochemical reaction in PBS (0.1 M, pH 7) are very similar in both species after extraction with water as the solvent. This does not mean that the electrochemically active substances in these two species are necessarily the same. Firstly, electrochemically active substances that are not soluble in water were not extracted. Secondly, some electrochemically active substances are also not involved in the reaction in the neutral electrolyte. Therefore, two solvents were selected, and electrochemical fingerprints were collected in two conditions. As shown in Figure 2, the fingerprints of *A. stachyophyllum* subsp. *betulifolium* and *A. cinnamomifolium* recorded after ethanol extraction in ABS (0.1 M, pH 4.5) showed a very distinct difference. *A. stachyophyllum* subsp. *betulifolium* has a distinct oxidation peak located at 0.37 V, while the *A. cinnamomifolium* have three oxidation in the range between 0.20–0.43 V. In addition, *A. stachyophyllum* subsp. *betulifolium* also exhibited a small oxidation at 0.06 V.

**Figure 3:**
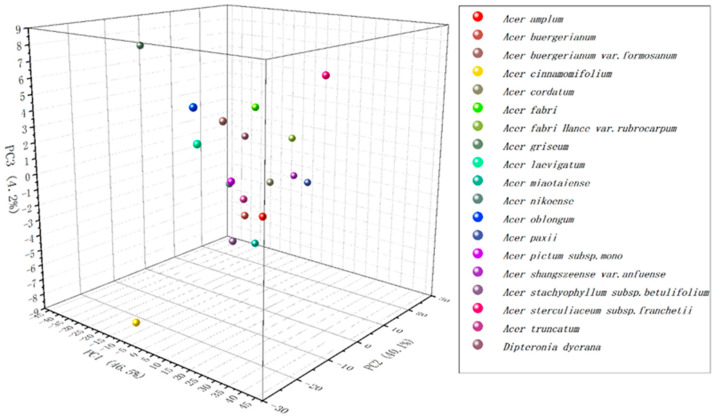
PCA analysis of *A. shangszeense* var. *anfuense*, *A. pictum* subsp. *mono*, *A. amplum*, *A. truncatum*, *A. miaotaiense*, *A. nikoense*, *A. griseum*, *A. fabri* Hance var. *rubrocarpum*, *A. paxii*, *A. oblongum*, *A. laevigatum*, *A. buergerianum* var. *formosanum*, *A. cinnamomifolium*, *A. buergerianum*, *A. cordatum*, *A. fabri*, *A. sterculiaceum* subsp. *Franchetii*, *A. stachyophyllum* subsp. *betulifolium*, and *Dipteronia dyerana* based on electrochemical fingerprints.

This work tried to statistically analyze all species using principal component analysis (PCA). PCA analysis is frequently used in phytochemical fingerprinting [14,18]. The electrochemical processes involve mainly solid-state reactions in the previous reports, where electron ingress is coupled, by reasons of charge conservation, to the entrance of protons on the solid lattice [18]. In this work, the fingerprints were recorded under solution rather than surface modification. Since the signal of the electrochemical fingerprint is able to respond to gene-level differences, statistical analysis can explain the similarity of electrochemically active substances between different species. As shown in Figure 3, after extracting three factors, PCA could reach 90.8% interpretation. This further indicated that electrochemical fingerprinting can be used for investigating the phylogenetic position of relatedness between different species.

**Figure 4:**
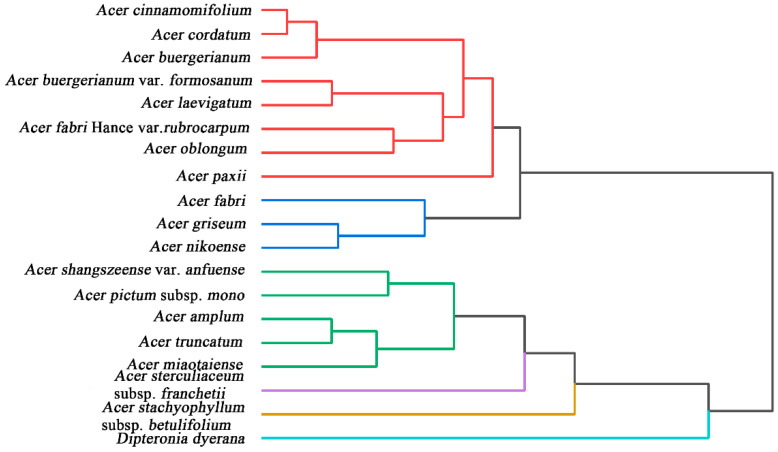
Dendrogram of *A. shangszeense* var. *anfuense*, *A. pictum* subsp. *mono*, *A. amplum*, *A. truncatum*, *A. miaotaiense*, *A. nikoense*, *A. griseum*, *A. fabri Hance* var. *rubrocarpum*, *A. paxii*, *A. oblongum*, *A. laevigatum*, *A. buergerianum* var. *formosanum*, *A. cinnamomifolium*, *A. buergerianum*, *A. cordatum*, *A. fabri*, *A. sterculiaceum* subsp. *Franchetii*, *A. stachyophyllum* subsp. *betulifolium*, and *Dipteronia dyerana* based on electrochemical fingerprints.

Aceraceae is the largest family of broad-leaved deciduous and evergreen forests distributed in a wide range of Asia, Eastern North America, and Europe. However, the genus *Acer* and *Dipteronia* have been placed in Aceraceae as both are found sharing numerous morphological characters. Recent studies have supported the close phylogenetic relationship between *Acer* and *Dipteronia* [42]. In our present work, *Dipteronia dyerana* was treated as an exo-taxa. The data used for the phylogenetic analysis were superimposed on the fingerprint profiles collected under both conditions. Based on the results in Figure 4, it can be seen that *Dipteronia dyerana* was not clustered with other species of *Acer*. Therefore, we believe that the interspecific affinities of *Acer* are still closer to *Dipteronia*. The entire phylogenetic tree of *Acer* is divided into three main clades. The first clade contains *A. cinnamomifolium*, *A. cordatum*, *A. buergerianum*, *A. buergerianum* var. *formosanum*, *A. laevigatum*, *A. fabri Hance* var. *rubrocarpum*, and *A. oblongum.* The second clade contains *A. fabri*, *A. nikoense*, and *A. griseum*. The third clade contains *A. shangszeense* var. *anfuense*, *A. pictum* subsp. *mono*, *A. amplum*, *A. truncatum*, and *A. miaotaiense*. All species can be divided into five subsections based on previous report [43]. Among them, *A. shangszeense* var. *anfuense*, *A. pictum* subsp. *mono*, *A. amplum*, *A. truncatum*, and *A. miaotaiense* belong to the subsection Platanoidea. This result is in full agreement with our clustering analysis based on electrochemical fingerprinting. *A. nikoense* and *A. griseum* belong to the subsection Trifoliata. The two species were indeed clustered together in the dendrogram, although *A. farbi* (belong to Integrifolia) also showed very similar affinities to them. It has also been previously reported that Integrifolia and Trifoliata are more closely related, and it is thought that they form the same evolutionary clade [44]. Their study concluded that the clusters formed by Integrifolia, Pentaphylla, and Trifoliata formed a sister taxon relationship with Hyptiocarpa. Another group that fits the traditional classification results is in the subsection Integrifolia. *A. cinnamomifolium*, *A. buergerianum*, *A. cordatum*, *A. laevigatum*, *A. buergerianum* var. *formosanum*, *A. fabri* Hance var. *rubrocarpum*, *A. paxii*, and *A. oblongum* all belong to Integrifolia. In addition, *A. sterculiaceum* subsp. *Franchetii* and *A. stachyophyllum* subsp. *betulifolium* belong to Lithocarpa and Arguta [45], respectively. They are not clustered together with other subsections.

## 4. Conclusions

In conclusion, the electrochemical fingerprint of the 18 species of *Acer* with an exo-taxa were recorded using water and ethanol extracts in PBS and ABS as supporting electrolytes. These electrochemical fingerprints can be used for phylogenetic investigation. *Dipteronia dyerana* was not clustered with other species of *Acer*. The interspecific affinities of *Acer* are still closer to *Dipteronia*. The phylogenetic tree of *Acer* is divided into three main clades. The result is in full agreement with *A. shangszeense* var. *anfuense*, *A. pictum* subsp. *mono*, *A. amplum*, *A. truncatum*, and *A. miaotaiense* belonging to the subsection Platanoidea. *A. nikoense* and *A. griseum* were clustered together in the dendrogram. Another group that fits the traditional classification results is in the subsection Integrifolia. Therefore, the electrochemical fingerprint recording provides an enormous potential for this method to become an assistive technology for future phylogenetic studies.

## Data Availability

Data sharing not applicable.
